# Case report: Rare novel *MIPEP* compound heterozygous variants presenting with hypertrophic cardiomyopathy, severe lactic acidosis and hypotonia in a Chinese infant

**DOI:** 10.3389/fcvm.2022.1095882

**Published:** 2023-01-16

**Authors:** Ling Wang, Pengtao Lu, Jie Yin, Kangkang Xu, Dandan Xiang, Zhongman Zhang, Han Zhang, Bixia Zheng, Wei Zhou, Chunli Wang, Shiwei Yang

**Affiliations:** ^1^Department of Cardiology, Children’s Hospital of Nanjing Medical University, Nanjing, China; ^2^Nanjing Key Laboratory of Pediatrics, Children’s Hospital of Nanjing Medical University, Nanjing, China

**Keywords:** *MIPEP*, hypertrophic cardiomyopathy, mitochondrial disease, oxidative phosphorylation, combined oxidative phosphorylation deficiency-31

## Abstract

**Background:**

Mitochondrial intermediate peptidase, encoded by the *MIPEP* gene, is involved in the processing of precursor mitochondrial proteins related to oxidative phosphorylation. Only a few studies have shown that mutations in *MIPEP* can cause combined oxidative phosphorylation deficiency-31 (COXPD31), an autosomal recessive multisystem disorder associated with mitochondrial dysfunction. We report herein a rare case of an 8-month-old boy in China with hypertrophic cardiomyopathy (HCM), severe lactic acidosis, and hypotonia caused by novel *MIPEP* compound heterozygous variants.

**Methods:**

Trio-whole-exome sequencing and copy number variation sequencing were performed to identify mutated genetic loci. Sanger sequencing and quantitative real-time PCR were used to validate the candidate single nucleotide variants and copy number variants, respectively.

**Results:**

The proband was an 8-month-old boy with HCM, severe lactic acidosis, and hypotonia who died 2 months after his first admission. Two novel compound heterozygous variants, c.1081T > A (p. Tyr361Asn) and a whole deletion (Ex1-19 del), were found in the *MIPEP* gene, which were inherited from his healthy parents respectively. Additionally, his mitochondria DNA copy number was significantly reduced.

**Conclusion:**

We are the first to report a patient with rare *MIPEP* variants in China. Our findings expand the mutation spectrum of *MIPEP*, and provide insights into the genotype-phenotype relationship in COXPD31.

## Introduction

Mitochondrial diseases (MDs) are rare, with a prevalence of 5–12/100,000 (1). They are characterized by oxidative phosphorylation (OXPHOS) dysfunction caused by nuclear and/or mitochondrial DNA (mtDNA) variations (2–4). Approximately 20–40% of children with MDs develop cardiac manifestations, such as hypertrophic cardiomyopathy (HCM), dilated cardiomyopathy (DCM), arrhythmias, left ventricular non-compaction (LVNC), heart failure and sudden cardiac death (5–8), which are termed mitochondrial cardiomyopathy (MCM) (2, 4). Combined oxidative phosphorylation deficiency-31 (COXPD31) (OMIM: 617228) is an autosomal recessive mitochondrial disease caused by mutations in the *MIPEP* gene. It can manifest as LVNC, HCM, DCM, global developmental delay, severe hypotonia, seizures, cataracts, and abnormal movements (9, 10). The *MIPEP* gene spans 57 kb in the long arm of chromosome 13 (13q12.12) and consists of 19 exons (11). Mitochondrial intermediate peptidase (MIP), encoded by *MIPEP*, which localizes to the mitochondrial matrix, and participates in secondary cleavage processing for a specific class of nuclear-encoded precursor mitochondrial proteins mostly characterized by XRX(↓)(F/L/I)XX(T/S/G)XXXX(↓) (11–13). Pulman et al. (10) demonstrated that *MIPEP* variants impair the stability and abundance of OXPHOS complexes. Clinical reports of *MIPEP* variations have been exceedingly infrequent. We herein reported the first case of early-onset HCM, severe lactic acidosis, and hypotonia, caused by *MIPEP* variants in China.

## Materials and methods

### Whole-exome sequencing

Genomic DNA was extracted from the peripheral blood of the proband and his parents using a DNA isolation kit (Tiangen, China) according to the manufacturer’s protocol. Following, the Genomic DNA was sheared into fragments and hybridized with the xGen Exome Research Panel v1.0 probe sequence capture array from IDT (Integrated Device Technology, USA) to enrich the exonic region. The enriched libraries were performed on an Illumina HiSeq XTen (Illumina, USA) platform. Variants with a minor allele frequency higher than 1% were filtered out. All identified variants were annotated using Genome Aggregation Database (gnomAD), 1000 Genomes Project (Chinese), dbSNP, and ExAC database. The candidate variants were further validated by Sanger sequencing and the pathogenicity of variants was evaluated according to the American College of Medical Genetics and Genomics (ACMG) criteria.

### Copy number variation calling

Copy number variations detection and annotation: We use CANOE, CNVnator, DeviCNV, and ExomeDepth to detect CNVs from WES data, and all CNVs were annotated to obtain additional information about the population frequencies and possible effects. The population frequencies for CNVs were obtained from Database of Genomic Variants (DGV). To assess the inclusion of any established dosage-sensitive genes or regions and the possible impact on gene function, each CNV was evaluated against a select set of haploinsufficient and triplosensitive genes and genomic regions obtained from ClinGen and Database of Chromosomal Imbalance and Phenotype in Humans Using Ensembl Resource (DECIPHER).

### Exon CNV analysis

The primer pair sequences are shown in Supplementary Table 1. Samples for quantitative real-time PCR(PT-qPCR) were assayed using the Takara SYBR Green with *ALB* genomic content used as an endogenous control for normalization of the data. The relative *MIPEP* gene expression was measured by subtracting the Ct values of the three exons (E1, E10, and E19) from the *ALB* gene, using the 2^–ΔΔCt^ method.

### Mitochondrial DNA (mtDNA) copy number assay

The genomic DNA was isolated from the whole blood of the proband and 3 normal controls, respectively. Then, the mean mtDNA copy number was determined by RT-qPCR using SYBR Green Real-Time PCR Master Mix (Takara, Japan) in a 10 μl reaction volume, including 1.6 μl of primers, 1.0 μl of DNA, 5 μl of 2 × Taq Master Mix (Vazyme Biotech Co., Ltd., Nanjing, China), and 2.4 μl of ddH_2_O. According to the instructions, the amplification cycles were as follows: 95°C for 30 s followed by 40 cycles of 95°C for 5 s, 60°C for 30 s and 72°C for 30 s. The melt curve stage includes 95°C for 10 s, 65°C for 5 s. By comparing the levels of mitochondrial DNA copy number (MT-ND2) versus nuclear DNA (18S), the relative levels of mtDNA copy numbers were assessed. Analyses were done in triplicates.

## Results

### Clinical presentation

**FIGURE 1:**
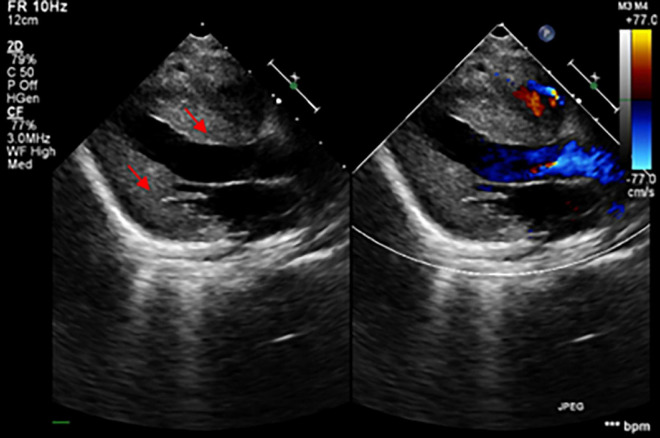
The echocardiogram demonstrated the thickening of the myocardium (the red arrows showed the hypertrophic interventricular septum and posterior wall of the left ventricle).

The patient was an 8-month-old male, the only child of healthy unrelated Chinese parents, born full-term after normal pregnancy and delivery. He was admitted due to light coma and poor response to external stimuli. His vital signs were as follows: body temperature, 37°C; heart rate, 180/min; respiratory rate, 45/min; and blood pressure, 75/45 mmHg. He presented with respiratory distress associated with severe lactic acidemia (lactate, 17.7 mmol/L; pH, 7.138), requiring mechanical ventilation. His psychomotor development was delayed, and he could not sit unaided at 8 months due to global hypotonia. Notably, he had a short penis and undescended testicles. Echocardiography revealed the features suggesting HCM (Figure 1). Specifically, the posterior left ventricle wall was slightly thickened (6 mm) and the interventricular septum was primarily thickened (13.2 mm) (Z-scores were 3.29 and 21.83, respectively),^1^ with mild left ventricular systolic dysfunction (left ventricular ejection fraction, 50%), there was minimal pericardial effusion with no abnormal valve morphology or motion. Brain magnetic resonance imaging showed abnormal signals on the bilateral thalamus and dorsal brainstem, strongly suggesting metabolic encephalopathy or inflammation. Furthermore, laboratory examinations showed markedly elevated plasma B-type natriuretic peptide (BNP) (2,613 pg/ml; upper limit of normal, 100 pg/ml) and slightly elevated liver enzyme levels [alanine transaminase (ALT), 121 U/L (reference, 0–41 U/L); aspartate transaminase (AST),105 U/L (reference,15–40 U/L)]. Urine creatinine and electrolyte levels were within normal ranges. Blood tests for genetic metabolic diseases revealed an abnormal increase in multiple acylcarnitine and 3-hydroxybutyrate levels. MD was then considered, and the patient was treated with high doses of coenzyme Q10, L-carnitine, vitamin B complex, etc. His blood PH value ultimately recovered to normal after a series of therapies, but lactic acid levels remained extraordinarily high (8–9 mmol/L). Finally, the boy’s condition worsened and died 2 months after admission. All his family members had no similar conditions.

### Genetic analysis

**FIGURE 2:**
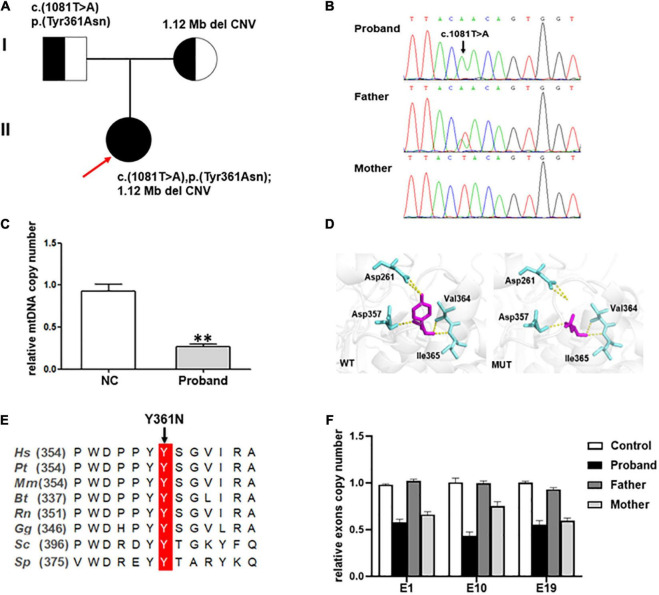
**(A)** Pedigree of the family reported in the present study. The arrow depicts the proband. The circles correspond to the women. The squares indicate the men. **(B)** Direct sequencing showing the novel missense variant c.1081T > A of the *MIPEP* gene. **(C)** The mtDNA copy numbers in healthy controls (*n* = 3) and the proband. A notable reduction in the mtDNA level was noted in the proband (NC, normal control, ^**^P < 0.01). **(D)** Structural visualization of the identified *MIPEP* missense alteration using Alpha Fold structure of the human *MIPEP* as a template (Identifier:AF-Q99797-F1). The variant site and the relevant residues were shown as sticks (the variant site is shown in magenta and the relevant residues are shown in aquamarine). The yellow dotted line represents the H-bond that connects the variant site with the residues. **(E)** Alignment of *MIPEP* orthologs in different species around the mutated amino acids residues. *Hs*, *Homo sapiens*; *Pt*, *Pan troglodytes; Mm*, *Macaca mulatta; Bt*, *Bos taurus; Rn*, *Rattus norvegicus; Gg*, *Gallus gallus; Sc*, *Saccharomyces cerevisiae S288C; Sp*, *Schizosaccharomyces pombe.* **(F)** *MIPEP* gene exons RT-qPCR analysis showed a heterozygous loss of exons 1, 10, and 19 in the proband and his mother.

Genetic analysis revealed that the proband had two compound-heterozygous variants in *MIPEP* (NM_005932): a hemizygous variant c.1081T > A (p. Tyr361Asn) and a 1.12-Mb deletion (chr13:23777833-24895906) containing the entire gene (Figures 2A, B), neither of which has been previously reported.

The mtDNA content and alterations were associated with OXPHOS complex deficiency-related disease, which are the biomarker of mitochondrial function and may reflect the degree of mtDNA damage (14). We examined the mtDNA copy number from whole blood samples to indirectly estimate the function of *MIPEP* variants. And the result showed that the relative ratio of mtDNA copy number of the proband was significantly decreased by 71.5%, compared to that in the healthy controls (*p* < 0.001; Figure 2C).

## Discussion

Here we reported a rare case with infantile-onset progressive cardiomyopathy and lactic acidosis. Genetic analysis identified a paternal missense variant c.1081T > A (p. Tyr361Asn) and a maternal hemizygous whole deletion (Ex1_19 del) in *MIPEP*, neither of which has been reported previously.

The c.1081T > A [p. (Tyr361Asn)] variant, absent from controls (1,000 Genomes, ExAC, gnomAD, and CNGB) (PM2), which was a missense variant in *MIPEP* involving a tyrosine-to-asparagine substitution at position 361. PolyPhen2, Mutation Taster and Provean predicted it as probably damaging, disease causing, and deleterious, respectively (PP3). According to ACMG, c.1081T > A (p. Tyr361Asn) was classified as a variant of uncertain significance (VUS) (PM2 + PP3). But the three-dimensional MIP molecular model showed this variant breaking its connection with aspartate at position 261 by H-bond, which destroyed the spatial structure of the protein (Figure 2D). Protein alignments also revealed the variant affected an amino acid highly conserved among species (Figure 2E). WES revealed that the patient and his mother may have a gene deletion (chr13:23777833-24895906), which involved eight genes (*MIPEP*, *SGCG*, *SACS*, *TNFRSF19*, *SPATA13*, *C1QTNF9*, *C1QTNF9B*-*AS1* and *C1QTNF9B*). Copy number analysis of three representative exons (E1, E10 and E19) was performed by RT-qPCR (Figure 2F) to verify the deletion detected by WES, and the results confirmed that the patient and his mother harbored the heterozygous deletion in *MIPEP* which was absent from controls (1000 Genomes, ExAC, gnomAD, and CNGB) (PM2). The deletion could have reduced MIP protein expression (PVS1), and it was classified as likely pathogenic (PVS1 + PM2) based on ACMG criteria. Moreover, the mtDNA copy number of the proband was significantly reduction comparing to that of healthy controls, suggesting that *MIPEP* may play a crucial role in regulating mitochondrial function. No variants in other MDs-related genes, such as *ACAD9*, *GTPBP3*, *NDUFV1*, *NCOA6*, *MMUT*, and *KARS1*, and the mtDNA were found in the proband. Therefore, the two variants were thought to cause the disease (15, 16).

To date, *MIPEP* variants have not been reported in Chinese patients. Only seven *MIPEP* variants in five patients have been reported worldwide, including five missense variants (p.Leu582Arg, p.Leu71Gln, p.Leu306Phe, p.Lys343Glu, p.His512Asp), one frameshift variant (p.Ala658Lysfs*38) and one deletion variant (1.4-Mb deletion, including the entire gene) (9, 10), all of which were inherited from their parents, following an autosomal recessive inheritance pattern. Four patients showed significant cardiac manifestations: 1 LVNC, 2 HCM, and 1 LVNC combined with DCM. Among them, three patients (75%) succumbed to progressive cardiac failure or sudden cardiac death before 2 years of age. Nevertheless, the patient with no cardiac symptoms who survived for more than 20 years (9, 10). Compared to the previous reports, the proband we reported had similar clinical presentations, such as cardiac abnormality, developmental delay, significant hypotonia, and lactic acidosis. Due to worsening condition even despite cocktail therapy, our patient died after 2 months treatment. The genetic and clinical information of all the patients are summarized in Table 1. These cases improved our awareness of COXPD31, and early recognition of MCM is essential to avoid heart failure and sudden cardiac death. Furthermore, identification of this severe early-onset condition expands the phenotypic spectrum associated with loss of MIP function, such as cardiomyopathy and other systemic impairments. Nowadays, most treatments for MCM could support and improve the quality of life to some extent. However, identifying an effective treatment modality remains difficult, owing to the heterogeneity of the disease.

**TABLE 1:** The *MIPEP* gene variants in six unrelated patients from six unrelated families.

References	This case	Eldomery et al. (9)	Eldomery et al. (9)	Eldomery et al. (9)	Eldomery et al. (9)	Pulman et al. (10)
Patient ID	P1	P2	P3	P4	P5	P6
Age of onset	8M	5.5M	11 M	5 M	At birth	8M
Variants	p.Y361N; loss1 (Exon:1-19)	p.L582R; p.L71Q	p.L306F; p.E602*	p.K343E	p.H512D; 1.4-Mb deletion	p.L306F; p.A658Kfs*38
History of pregnancy and delivery	N	N	N	36 weeks gestation due to preterm labor	33 weeks gestation due to maternal preeclampsia and fetal decelerations	N
Family history	The farther with a history of tuberculous pleurisy	A paternal uncle with a history of supra-ventricular tachycardia and maternal great-aunt with early myocardial infarction (29 years of age)	An older brother had cataracts and infantile spasms and died unexpectedly at 14 months of age of unknown cause	The parents are first-degree cousins	His sister presented with cardiomyopathy in the immediate postnatal period and subsequently expired by 16 days of life	N
Electrocardiogram	N	Wolf-Parkinson-White syndrome	N	U	U	N
Echocardiography	HCM	LVNC	LVNC and DCM	HCM	HCM	N
Lactic acid (mmol/L)	17.7	3.2	U	11.1	8.9–10.4	2.2
Other features	Short penis, the testicles did not descend into the scrotum, could not sit by himself and had significant hypotonia	Wide mouth and bulbous nasal tip, tongue-thrusting, hypotonia with head lag, abnormal movements and dystonic posturing	Cataract, hypotonia, developmental delay and uncontrollable seizures	Long philtrum, opisthotonus and severe head lag when pulled to sit, microcephaly and seizures	Deep-set eyes, anteverted nares, depressed nasal bridge, midface hypoplasia, severe micrognathia, facial asymmetry, and an accessory palmar crease on the right hand	Developmental delay, global hypotonia, mild optic neuropathy and mild ataxia
Outcome	Died at 10 months	Alive at the age of 4.5 years old	Died at 2 years old	Died at 11 months	Died at 19 days	Alive at the age of 20 years old

P, patient; M, month; N, normal; U, unclear; HCM, hypertrophic cardiomyopathy; LVNC, left ventricular non-compaction; DCM, dilated cardiomyopathy.

## Conclusion

We are the first to report a rare case of an 8-month-old boy in China with *MIPEP* variations who presented with HCM, severe lactic acidosis, and hypotonia. Genetic analysis revealed novel compound heterozygous variants c.1081T > A [p. (Tyr361Asn)] and a whole deletion (Ex1_19 del) in the *MIPEP* gene. Our findings expand the genetic spectrum of *MIPEP*-linked mitochondrial disease, and highlight the importance of an interrelationship between clinical and research for the identification of disease-associated genes.

## Data availability statement

The original contributions presented in this study are included in the article/Supplementary material, further inquiries can be directed to the corresponding author.

## Ethics statement

The studies involving human participants were reviewed and approved by the Institutional Ethical Committee of the Children’s Hospital of Nanjing Medical University. Written informed consent to participate in this study was provided by the participants’ legal guardian/next of kin. Written informed consent was obtained from the individual(s), and minor(s)’ legal guardian/next of kin, for the publication of any potentially identifiable images or data included in this article.

## Author contributions

LW edited the manuscript. PL contributed samples collection. JY and SY revised the manuscript. All authors contributed to the article and approved the submitted version.

## Footnotes

1
http://zscore.chboston.org

## References

[1] Mazzaccara C Mirra B Barretta F Caiazza M Lombardo B Scudiero O Molecular epidemiology of mitochondrial cardiomyopathy: a search among mitochondrial and nuclear genes. *Int J Mol Sci.* (2021) 22:5742. 10.3390/ijms22115742 34072184 PMC8197938

[2] Imai-Okazaki A Kishita Y Kohda M Mizuno Y Fushimi T Matsunaga A Cardiomyopathy in children with mitochondrial disease: prognosis and genetic background. *Int J Cardiol.* (2019) 279:115–21. 10.1016/j.ijcard.2019.01.017 30642647

[3] Kohda M Tokuzawa Y Kishita Y Nyuzuki H Moriyama Y Mizuno Y A comprehensive genomic analysis reveals the genetic landscape of mitochondrial respiratory chain complex deficiencies. *PLoS Genet.* (2016) 12:e1005679. 10.1371/journal.pgen.1005679 PMC4704781 26741492

[4] Tokuyama T Ahmed RE Chanthra N Anzai T Uosaki H. Disease modeling of mitochondrial cardiomyopathy using patient-specific induced pluripotent stem cells. *Biology.* (2021) 10:981. 10.3390/biology10100981 34681080 PMC8533352

[5] Meyers DE Basha HI Koenig MK. Mitochondrial cardiomyopathy: pathophysiology, diagnosis, and management. *Tex Heart Inst J.* (2013) 40:385–94. 24082366 PMC3783139

[6] El-Hattab AW Scaglia F. Mitochondrial cardiomyopathies. *Front Cardiovasc Med.* (2016) 3:25. 10.3389/fcvm.2016.00025 27504452 PMC4958622

[7] Brunel-Guitton C Levtova A Sasarman F. Mitochondrial diseases and cardiomyopathies. *Can J Cardiol.* (2015) 31:1360–76. 10.1016/j.cjca.2015.08.017 26518446

[8] Limongelli G Masarone D Pacileo G. Mitochondrial disease and the heart. *Heart.* (2017) 103:390–8. 10.1136/heartjnl-2015-308193 27821705

[9] Eldomery MK Akdemir ZC Vogtle FN Charng WL Mulica P Rosenfeld JA MIPEP recessive variants cause a syndrome of left ventricular non-compaction, hypotonia, and infantile death. *Genome Med.* (2016) 8:106. 10.1186/s13073-016-0360-6 PMC5088683 27799064

[10] Pulman J Ruzzenente B Horak M Barcia G Boddaert N Munnich A Variants in the MIPEP gene presenting with complex neurological phenotype without cardiomyopathy, impair OXPHOS protein maturation and lead to a reduced OXPHOS abundance in patient cells. *Mol Genet Metab.* (2021) 134:267–73. 10.1016/j.ymgme.2021.09.005 34620555

[11] Chew A Buck EA Peretz S Sirugo G Rinaldo P Isaya G. Cloning, expression, and chromosomal assignment of the human mitochondrial intermediate peptidase gene (MIPEP). *Genomics.* (1997) 40:493–6. 10.1006/geno.1996.4586 9073519

[12] Hendrick JP Hodges PE Rosenberg LE. Survey of amino-terminal proteolytic cleavage sites in mitochondrial precursor proteins: leader peptides cleaved by two matrix proteases share a three-amino acid motif. *Proc Natl Acad Sci U.S.A.* (1989) 86:4056–60. 10.1073/pnas.86.11.4056 2657736 PMC287387

[13] Coenen MJ Smeitink JA Smeets R Trijbels FJ van den Heuvel LP. Mutation detection in four candidate genes (OXA1L, MRS2L, YME1L and MIPEP) for combined deficiencies in the oxidative phosphorylation system. *J Inherit Metab Dis.* (2005) 28:1091–7. 10.1007/s10545-005-4483-y 16435202

[14] Medeiros DM. Assessing mitochondria biogenesis. *Methods.* (2008) 46:288–94. 10.1016/j.ymeth.2008.09.026 18929661

[15] Bernier FP Boneh A Dennett X Chow CW Cleary MA Thorburn DR. Diagnostic criteria for respiratory chain disorders in adults and children. *Neurology.* (2002) 59:1406–11. 10.1212/01.wnl.0000033795.17156.00 12427892

[16] Klopstock T Priglinger C Yilmaz A Kornblum C Distelmaier F Prokisch H. Mitochondrial disorders. *Dtsch Arztebl Int.* (2021) 118:741–8. 10.3238/arztebl.m2021.0251 34158150 PMC8830351

